# PARP3 Promotes SARS-CoV-2 Replication via Interaction with the Viral Nucleocapsid Protein

**DOI:** 10.24546/0100505590

**Published:** 2026-08-31

**Authors:** DEWA NYOMAN MURTI ADYAKSA, AULIA FITRI RHAMADIANTI, LIN DENG, TAKAYUKI ABE, CHIEKO MATSUI, IKUO SHOJI

**Affiliations:** 1 Division of Infectious Disease Control, Center for Infectious Diseases, Kobe University Graduate School of Medicine, Kobe, Japan; 2 Department of Anatomical Pathology, Faculty of Medicine, Public Health and Nursing, Gadjah Mada University, Yogyakarta, Indonesia; 3 Department of Virology, Niigata University Graduate School of Medical and Dental Sciences, Niigata, Japan

**Keywords:** SARS-CoV-2, Nucleocapsid, PARP3, Venadaparib, Antiviral

## Abstract

Severe acute respiratory syndrome coronavirus 2 (SARS-CoV-2) exploits host cellular machinery to support its replication. Members of the host poly(ADP-ribose) polymerase (PARP) family regulate the DNA damage response and have been implicated in both antiviral and proviral processes. In this study, we investigated the potential role of PARP3 in SARS-CoV-2 replication in VeroE6/Rep3 cells. siRNA-mediated knockdown of PARP3 reduced viral subgenomic RNA (sgRNA) levels and reporter activity, whereas PARP3 overexpression enhanced these processes, suggesting that PARP3 promotes SARS-CoV-2 replication. In addition, PARP3 expression was increased in SARS-CoV-2-infected Calu-3 cells and in cells expressing SARS-CoV-2 nucleocapsid protein, suggesting that the nucleocapsid protein contributes to PARP3 upregulation during infection. Co-immunoprecipitation and immunofluorescence analyses revealed that PARP3 associates with the nucleocapsid protein and redistributes to the cytoplasm in nucleocapsid-expressing cells. Screening of a PARP inhibitor library identified venadaparib as a compound that reduced SARS-CoV-2 sgRNA levels and reporter activity in VeroE6/Rep3 cells with limited cytotoxicity under the condition tested. We further found that venadaparib reduced the interaction between PARP3 and the nucleocapsid protein. Molecular docking analysis further suggested that venadaparib can bind to the PARP3 catalytic domain. Collectively, these findings support a model in which PARP3 facilitates SARS-CoV-2 replication through its interaction with the viral nucleocapsid protein and suggest that venadaparib warrants further evaluation as a host-directed antiviral candidate.

## INTRODUCTION

Severe acute respiratory syndrome coronavirus 2 (SARS-CoV-2) utilizes host intracellular networks to complete its replicative cycle (1). Upon viral entry, the viral genome is translated into non-structural proteins that remodel host endomembranes to form double-membrane vesicles, which serve as sites for viral replication and transcription (2). Concurrently, the structural nucleocapsid (N) protein mediates the encapsidation of nascent viral RNA (2, 3). The N protein functions as a multifunctional viral protein that interacts with host signaling pathways to modulate cellular metabolism and evade innate immunity (4).

Viral replication perturbs the host DNA damage response (DDR) (5). Emerging clinical and experimental evidence indicates that SARS-CoV-2 replication triggers profound cellular stress, leading to host DNA damage. Pathological profiling of pulmonary tissue derived from fatal COVID-19 cases has demonstrated that severe oxidative stress and the hyperactivation of the host poly(ADP-ribose) polymerase (PARP) system are early hallmarks of COVID-19 pneumonia (6). The human PARP family consists of 17 isozymes that regulate DDR, chromatin dynamics, and innate immune signaling by catalyzing the transfer of ADP-ribose units to target proteins (7). Several PARPs are involved in viral infections, acting either as antiviral factors or as proviral host factors. For example, PARP1 overactivation facilitates the replication of influenza A virus and Japanese encephalitis virus (8, 9). Accordingly, the PARP inhibitors stenoparib and rucaparib have demonstrated preclinical efficacy as host-directed antivirals against SARS-CoV-2 (10–12). Despite these findings, the precise mechanisms by which host PARP family members regulate SARS-CoV-2 replication remain incompletely understood.

PARP3 is a mono-ADP-ribosyltransferase primarily recognized as a nuclear enzyme that contributes to double-strand break repair through non-homologous end joining (NHEJ) (13). However, its role in RNA virus replication remains poorly defined. Transcriptomic data from SARS-CoV-2-infected human lung epithelial cells (GSE147507) indicate upregulation of PARP transcripts, suggesting their potential involvement in the viral life cycle (14, 15).

In the present study, we investigated the role of PARP3 in SARS-CoV-2 replication. We found that PARP3 promoted viral replication and that SARS-CoV-2 infection was associated with enhanced PARP3 expression. Moreover, PARP3 associated with the viral N protein in the cytoplasm. We also identified venadaparib, a selective PARP inhibitor (16), as a compound that reduced SARS-CoV-2 sgRNA levels and reporter activity . We also found that venadaparib reduced the interaction between nucleocapsid and PARP3. These findings suggest that PARP3 exerts a proviral effect through its interaction with the N protein and suggest that the PARP3–N protein axis represents a potential host-directed therapeutic target in SARS-CoV-2 infection.

## MATERIALS AND METHODS

### Cell cultures

HEK293T cells were maintained in Dulbecco’s modified Eagle’s medium (DMEM; high glucose) with L-glutamine (Fujifilm Wako Pure Chemical Industries, Osaka, Japan) supplemented with 50 IU/mL penicillin, 50 μg/mL streptomycin (Gibco, Grand Island, NY, USA), and 10% heat-inactivated fetal bovine serum (FBS) (Biowest, Nuaillé, France) at 37°C in a 5% CO_2_ incubator. The SARS-CoV-2 replicon cells (VeroE6/Rep3) were reported previously (17). VeroE6/Rep3 cells were cultured in DMEM supplemented with 50 IU/mL penicillin, 50 μg/mL streptomycin, 10% FBS, 1% sodium pyruvate (Sigma-Aldrich, St. Louis, MO, USA), and 1 mg/mL G418 (Nacalai Tesque, Kyoto, Japan). Cells were transfected with plasmid DNA using TransIT reagents (Mirus Bio, Madison, WI, USA).

### Plasmid construction

Full-length human cDNA sequences encoding PARP1, PARP2, and PARP3 were amplified by high-fidelity PCR using Tks DNA Polymerase (TaKaRa Bio, Shiga, Japan). Purified amplicons were directionally cloned into the EcoRI site of the eukaryotic pCAG mammalian expression vector modified to include an N-terminal FLAG epitope tag, using the In-Fusion HD Cloning system (Clontech, Mountain View, CA, USA). The expression plasmid pCAG-HA-nucleocapsid was previously described (18).

### Antibodies and reagents

The mouse monoclonal antibodies (MAbs) used in this study were anti-PARP1 MAb (sc-8007; Santa Cruz Biotechnology, Santa Cruz, CA, USA), anti-PARP2 MAb (sc-393343; Santa Cruz Biotechnology), anti-PARP3 MAb (sc-390758; Santa Cruz Biotechnology), anti-FLAG (M2) mAb (F-3165; Sigma-Aldrich), and anti-GAPDH mAb (MAB374; Millipore, Billerica, MA, USA). The rabbit MAb used in this study was anti-nucleocapsid MAb (HL448) (GTX635686; GeneTex, Irvine, CA, USA). The rabbit polyclonal antibody (PAb) used in this study was anti-HA PAb (H-6908; Sigma-Aldrich). Horseradish peroxidase (HRP)-conjugated anti-mouse IgG (Cell Signaling Technology, Danvers, MA, USA) and HRP-conjugated anti-rabbit IgG (Cell Signaling Technology) were used as secondary antibodies.

Favipiravir and 38 PARP inhibitors, including venadaparib, were purchased from Selleck Chemicals (Houston, TX, USA).

### RNA interference

Target-specific siRNAs against human PARP1 (si-PARP1 #1: 5′-GGAGGAAGGUGUCAACAAA-3′, si-PARP1 #2: 5′-AACUCCGAGUCUCAGCCAA-3′), PARP2 (si-PARP2 #1: 5′-GCACGUGUCCAAGAGGCUA-3′, si-PARP2 #2: 5′-GAAAGGCCUUAUCGAUAAA-3′), and PARP3 (si-PARP3 #1: 5′-GGCAAAGAGCACCACAUCA-3′, si-PARP3 #2: 5′-GCAAGGAGCUCUGCUACAA-3′) as well as a scrambled non-targeting negative control (siRNA NC) were synthesized (Nippon Gene, Tokyo, Japan). VeroE6/Rep3 cells were transfected with 50 nM siRNA using RNAiMax transfection reagent (Life Technologies, Carlsbad, CA, USA) according to the manufacturer’s instructions.

### Immunoprecipitation and immunoblot analysis

Cultured cells were lysed with a buffer containing 50 mM Tris-HCl (pH 8.0), 150 mM NaCl, 0.1% SDS, 1% NP-40, 0.5% deoxycholate, and protease inhibitor cocktail tablets (Roche Molecular Biochemicals, Mannheim, Germany). Cell lysates were incubated for 2 h at 4°C and centrifuged at 20,400 × g for 30 min at 4°C. The supernatant was incubated with the appropriate antibodies at 4°C overnight and immunoprecipitated with protein G-Sepharose 4 Fast Flow (GE Healthcare, Buckinghamshire, UK). After being washed five times with lysis buffer, the samples were boiled in SDS sample buffer, subjected to SDS-polyacrylamide gel electrophoresis (SDS-PAGE), and transferred to polyvinylidene difluoride (PVDF) membranes (Millipore). The membranes were blocked with Tris-buffered saline containing 20 mM Tris-HCl (pH 7.6), 135 mM NaCl, and 0.05% Tween 20 (TBST) supplemented with 5% skim milk at room temperature for 2 h, and incubated with the corresponding antibodies. The membranes were then incubated with HRP-conjugated secondary antibodies at room temperature for 2 h. Immune complexes and cell lysates were visualized with ECL Western blotting detection reagents (GE Healthcare) and detected using an Amersham ImageQuant 800 system (GE Healthcare). Band intensities were quantified using ImageJ software version 1.56d.

### Luciferase assay

Luciferase activity was measured using the Renilla luciferase assay system (Promega, Madison, WI, USA) according to the manufacturer’s instructions. Cells were harvested at 72 h of incubation, solubilized in 1 × passive lysis buffer, and measured using a Glomax Navigator Microplate Luminometer GM2000 (Promega).

### Cell viability assay

The effect of venadaparib on cell viability was evaluated using the CellTiter-Glo 2.0 assay system (Promega) according to the manufacturer’s instructions. Cells were subjected to CellTiter-Glo analysis 5 days after compound treatment and measured using a Glomax® Navigator Microplate Luminometer GM2000 (Promega).

### RNA extraction and quantitative reverse transcription-PCR (RT-qPCR)

Total RNA was prepared using the RNeasy mini kit (Qiagen, Valencia, CA, USA), followed by cDNA synthesis using the GoScript reverse transcription system (Promega). PCR was performed using SYBR Premix Ex Taq II (Tli RNaseH Plus) (TaKaRa Bio, Shiga, Japan) according to the manufacturer’s instructions. Fluorescent signals were analyzed using a StepOne Plus real-time PCR system (Applied Biosystems, Foster City, CA, USA). The sgRNA and GAPDH genes were amplified using the following specific primer pairs: sgRNA forward, 5′-CGATCTCTTGTAGATCTGTTCTCT-3′; sgRNA reverse, 5′-GTCTTCCTTGCCATGTTGAGT-3′; GAPDH forward, 5′-GCCATCAATGACCCCTTCATT-3′; GAPDH reverse, 5′-TCTCGCTCCTGGAAGATGG-3′. Gene expression levels were determined using the ΔΔCt method with GAPDH as an internal control.

### Immunofluorescence assay

Cells were cultured on glass coverslips, fixed with 4% paraformaldehyde, permeabilized with 0.2% Triton X-100, and blocked with 2% BSA. Coverslips were probed with anti-HA PAb (H-6908; Sigma-Aldrich) and anti-FLAG MAb (F-3165; Sigma-Aldrich), followed by Alexa Fluor 488-conjugated goat anti-rabbit IgG (Molecular Probes, Eugene, OR, USA) and Alexa Fluor 594-conjugated goat anti-mouse IgG (Molecular Probes). Nuclei were counterstained with Hoechst 33342 (Molecular Probes). Stained cells were observed using a BZ-9000E fluorescence microscope (Keyence, Osaka, Japan).

### *In silico* transcriptomic analysis and molecular docking

*In silico* transcriptomic evaluation was conducted using RNA-sequencing data from human lung Calu-3 cells (GSE147507), comparing mock-infected and SARS-CoV-2-infected cells. Binding affinity prediction of venadaparib against the human PARP3 catalytic domain was performed using the high-resolution crystal structure (PDB ID: 4L70). The three-dimensional conformation of venadaparib within the nicotinamide-binding cleft was computed using the generative diffusion molecular docking algorithm (DiffDock) (19). Absolute binding energy (Δ*G*) and predicted binding affinity (p*Kd*) were evaluated using the GNINA scoring algorithm, which combines conventional physics-based empirical scoring with convolutional neural networks (20). Sub-pocket geometry and steric ligand coverage were analyzed using DoGSite3 (21) via the ProteinPlus server (22).

### Ethics statement

This study used established cell lines and publicly available transcriptomic data (GSE147507). No human participants or animals were directly involved; therefore, institutional review board approval and informed consent were not required.

### Statistical Analysis

Data are presented as the mean ± standard error of the mean (SEM) from at least three independent experiments. Comparisons between two groups were performed using an independent Student’s *t*-test. A P value < 0.05 was considered statistically significant.

## RESULTS

### PARP3 promotes SARS-CoV-2 replicon replication in VeroE6/Rep3 cells

To investigate the role of PARP family members in SARS-CoV-2 replication, we depleted PARP1, PARP2, and PARP3 using specific siRNAs in VeroE6/Rep3 cells, a SARS-CoV-2 reporter replicon cell line harboring replicon RNA encoding *Renilla* luciferase to monitor viral replication. Knockdown of PARP1, PARP2, and PARP3 each reduced viral subgenomic RNA (sgRNA) levels, with the strongest suppressive effect observed following PARP3 depletion (Fig. 1A, left panel). Similarly, the *Renilla* luciferase assay showed that depletion of each PARP reduced reporter activity, and this reduction was most pronounced after PARP3 knockdown (Fig. 1B). Immunoblot analysis further revealed that knockdowns of PARP1, PARP2, and PARP3 decreased viral nucleocapsid protein levels, with PARP3 knockdown producing the most marked reduction (Fig. 1C, top panel, lanes 2–4). Conversely, exogenous expression of FLAG-tagged PARP3 significantly enhanced both sgRNA levels (Fig. 1D) and reporter activity (Fig. 1E) in VeroE6/Rep3 cells. Because PARP3 showed the most pronounced effect among the PARP family members examined, we focused subsequent analyses on PARP3. These results suggest that PARP3 promotes SARS-CoV-2 replicon replication in VeroE6/Rep3 cells.

### SARS-CoV-2 nucleocapsid enhances PARP3 expression

To investigate whether PARP3 expression is altered during SARS-CoV-2 infection, we re-analyzed a bulk RNA-sequencing dataset (GSE147507) derived from human Calu-3 lung epithelial cells. Following normalization to counts per million (CPM), differential expression analysis revealed statistically significant upregulation of host PARP3 transcripts after infection compared with mock controls (Fig. 2A). These findings suggest that PARP3 may participate in host cellular responses to SARS-CoV-2 infection.

To determine whether the viral N protein affects PARP expression, HEK293T cells were transfected with pCAG-HA-nucleocapsid. RT-qPCR showed that expression of the SARS-CoV-2 N protein significantly increased endogenous PARP3 mRNA levels, whereas no comparable increase was observed for PARP1 or PARP2 (Fig. 2B). Immunoblot analyses further showed that N protein expression increased PARP3 protein levels (Fig. 2C, fourth panel), but not PARP1 (Fig. 2C, second panel) or PARP2 (Fig. 2C, third panel). These findings suggest a relatively selective effect of the N protein on PARP3 expression and indicate that the SARS-CoV-2 N protein may contribute to PARP3 upregulation during infection.

### SARS-CoV-2 N protein associates with PARP1, PARP2, and PARP3

To investigate whether the N protein physically associates with PARP1, PARP2, or PARP3, we performed co-immunoprecipitation analyses. HEK293T cells were co-transfected with pCAG-HA-nucleocapsid and either pCAG-FLAG-PARP1, pCAG-FLAG-PARP2, or pCAG-FLAG-PARP3. At 48 h after transfection, cells were harvested, and lysates were immunoprecipitated with anti-FLAG beads. Immunoprecipitation analysis showed that HA-nucleocapsid was co-immunoprecipitated with FLAG-PARP1 (Fig. 3A, top panel, lane 3), FLAG-PARP2 (Fig. 3B, top panel, lane 3), and FLAG-PARP3 (Fig. 3C, top panel, lane 3). To further confirm these associations, reciprocal immunoprecipitation assays were performed using anti-HA antibody. FLAG-PARP1 (Fig. 3D, top panel, lane 3), FLAG-PARP2 (Fig. 3E, top panel, lane 3), and FLAG-PARP3 (Fig. 3F, top panel, lane 3) were co-immunoprecipitated with HA-nucleocapsid. These results indicate that SARS-CoV-2 N protein associates with PARP1, PARP2, and PARP3.

### SARS-CoV-2 nucleocapsid colocalizes with PARP3 predominantly in the cytoplasm

To examine the subcellular localization of nucleocapsid and PARP3, we performed immunofluorescence staining. HEK293T cells were co-transfected with pCAG-FLAG-PARP3 and either pCAG-HA-nucleocapsid or an empty vector. When expressed alone, FLAG-PARP3 was predominantly localized in the nucleus (Fig. 4, upper panel, FLAG). When HA-nucleocapsid and FLAG-PARP3 were co-expressed, FLAG-PARP3 was predominantly redistributed to the cytoplasm (Fig. 4, lower panel, FLAG), and the merged yellow signals indicated that HA-nucleocapsid and FLAG-PARP3 colocalized predominantly in the cytoplasm (Fig. 4, lower panel, Merge). These results suggest that SARS-CoV-2 N protein may alter the subcellular localization of PARP3, resulting in cytoplasmic colocalization of nucleocapsid and PARP3 in the cytoplasm.

### Screening of a PARP inhibitor library identifies compounds with anti-SARS-CoV-2 activity

To investigate whether PARP upregulation could be therapeutically targeted, we evaluated a library of 38 PARP inhibitors (Table I). Among the compounds tested at 10 μM, niraparib (No. 1), veliparib (No. 9), and venadaparib (No. 36) significantly reduced sgRNA levels (Fig. 5B) and reporter activity (Fig. 5C) in VeroE6/Rep3 cells without marked cytotoxicity (Fig. 5A) under the experimental conditions used. Because venadaparib showed a robust inhibitory effect in this assay system, we selected it for further mechanistic analysis.

### Venadaparib suppresses SARS-CoV-2 replicon replication and reduces N protein expression

To further evaluate the antiviral effects of niraparib (No. 1), veliparib (No. 9), and venadaparib (No. 36), VeroE6/Rep3 cells were treated with 0.1, 1, or 10 μM of each compound for 72 h. We found that venadaparib significantly reduced both sgRNA levels (Fig. 6A) and reporter activity (Fig. 6B) across the concentration range tested, including the lowest concentration examined. In addition, immunoblotting analysis showed that venadaparib reduced nucleocapsid protein levels in a dose-dependent manner (Fig. 6C, top panel, lanes 2–4). These results suggest that venadaparib decreases SARS-CoV-2 replicon replication and decreases nucleocapsid expression under the experimental conditions used.

### Venadaparib reduces the association between PARP3 and nucleocapsid and is predicted to bind to the PARP3 catalytic domain

To investigate whether the antiviral activity of venadaparib is associated with reduced interaction between PARP3 and the N protein, we performed co-immunoprecipitation analyses. HEK293T cells were co-transfected with pCAG-FLAG-PARP3 and pCAG-HA-nucleocapsid. At 24 h after transfection, cells were treated with 1 μM niraparib (No. 1), veliparib (No. 9), or venadaparib (No. 36). At 72 h after treatment, cells were harvested. Co-immunoprecipitation assays showed that niraparib and veliparib slightly reduced the detectable association between HA-nucleocapsid and FLAG-PARP3 (Fig. 7A, top panel, lanes 2, 3), whereas venadaparib produced the greatest reduction under the same experimental conditions (Fig. 7A, top panel, lane 4). These data suggest that venadaparib may interfere with the PARP3-N protein association.

To explore the structural basis for the disruption of the PARP3-nucleocapsid interaction, we performed molecular docking of venadaparib against the human PARP3 catalytic domain (PDB ID: 4L70) using DiffDock. Subsequent deep-learning binding affinity evaluation using GNINA yielded a favorable binding affinity of −8.81 kcal/mol. The predicted three-dimensional complex revealed that the phthalazinone core of venadaparib occupies the nicotinamide-binding cleft and forms hydrogen bonds with the conserved residues Gly385 and Ser422 (Fig. 7B). In this conformation, venadaparib occupied 53.33% of the druggable subpocket volume predicted independently by DoGSite3.

## DISCUSSION

In this study, our data support the idea that host PARP3 acted as a proviral factor in SARS-CoV-2 replication (Fig. 1). In a SARS-CoV-2 replicon cell system, PARP3 depletion reduced sgRNA levels and reporter activity, whereas PARP3 overexpression enhanced these readouts. In addition, transcriptomic analysis showed that PARP3 expression was upregulated in SARS-CoV-2-infected cells (Fig. 2A), and the viral N protein physically interacted with PARP3 in the cytoplasm in our experimental systems (Figs. 3, 4). Importantly, we identified venadaparib as a compound that reduced SARS-CoV-2 replicon replication and reduced the association between PARP3 and the N protein (Figs. 5–7). Collectively, these findings support a model in which PARP3 promotes SARS-CoV-2 replication, potentially through association with the viral N protein, and that venadaparib represents a potential antiviral activity against SARS-CoV-2 (Fig. 8).

Previous studies have demonstrated that PARP activation and oxidative stress are associated with SARS-CoV-2 infection and COVID-19 pathogenesis (6). In agreement with these observations, we found PARP3 depletion significantly reduced sgRNA levels and reporter activity in VeroE6/Rep3 cells (Fig. 1). Furthermore, transcriptomic analysis of SARS-CoV-2-infected Calu-3 cells revealed upregulation of PARP3 expression (Fig. 2A), suggesting that SARS-CoV-2 infection modulates host DNA damage response pathways. Although the mechanism underlying PARP3 upregulation remains unclear, our findings suggest that PARP3 contributes to efficient SARS-CoV-2 replication.

A notable finding of this study is that the viral N protein associated with PARP3 and promoted its cytoplasmic redistribution in our experimental system (Figs. 3, 4). PARP3 is predominantly localized in the nucleus under basal conditions and is known to participate in DNA damage repair. Because SARS-CoV-2 replication takes place in the cytoplasm, this observation raises the possibility that altered subcellular localization of PARP3 may contribute to its proviral effect. The mechanism underlying this redistribution remains unclear. One possibility is that SARS-CoV-2 infection or nucleocapsid expression promotes the cytoplasmic accumulation of PARP3, thereby facilitating its interaction with the viral nucleocapsid and supporting viral replication. Further studies are needed to elucidate the molecular mechanisms regulating PARP3 localization during infection.

The molecular mechanism by which PARP3 promotes SARS-CoV-2 replication remains to be clarified. ISGylation, the covalent conjugation of interferon-stimulated gene 15 (ISG15) to target proteins, is an important antiviral defense mechanism (23). We previously reported that ISGylation of the SARS-CoV-2 N protein disrupts its oligomerization and suppresses viral replication, whereas the viral papain-like protease counteracts this restriction by inhibiting N protein ISGylation (18). On the basis of these findings, it is possible that PARP3 may influence pathways linked to N protein regulation. However, the present study was not designed to directly define the relationship between PARP3 and N protein ISGylation. Additional experiments will therefore be required to test this possibility.

Venadaparib was identified in our system as a compound that reduced SARS-CoV-2 sgRNA levels and reporter activity in VeroE6/Rep3 cells (Fig. 5). Molecular docking analysis further suggested that venadaparib may bind to the PARP3 catalytic domain (Fig. 7B). Although the precise mechanism by which venadaparib reduces the PARP3–N protein association remains unclear, the present findings are consistent with the possibility that venadaparib acts, at least in part, through PARP3-related mechanisms. Further studies will be needed to define its precise mode of action and therapeutic potential.

Collectively, these findings support a working model in which PARP3 associates with the viral N protein and contributes to SARS-CoV-2 replication (Fig. 8). SARS-CoV-2 infection promotes the cytoplasmic localization of PARP3, allowing PARP3 to interact with the viral nucleocapsid and facilitate viral replication. This proviral pathway is inhibited by venadaparib, which reduces the association between PARP3 and the N protein, thereby potentially suppressing SARS-CoV-2 replication.

## Figures and Tables

**Fig. 1:**
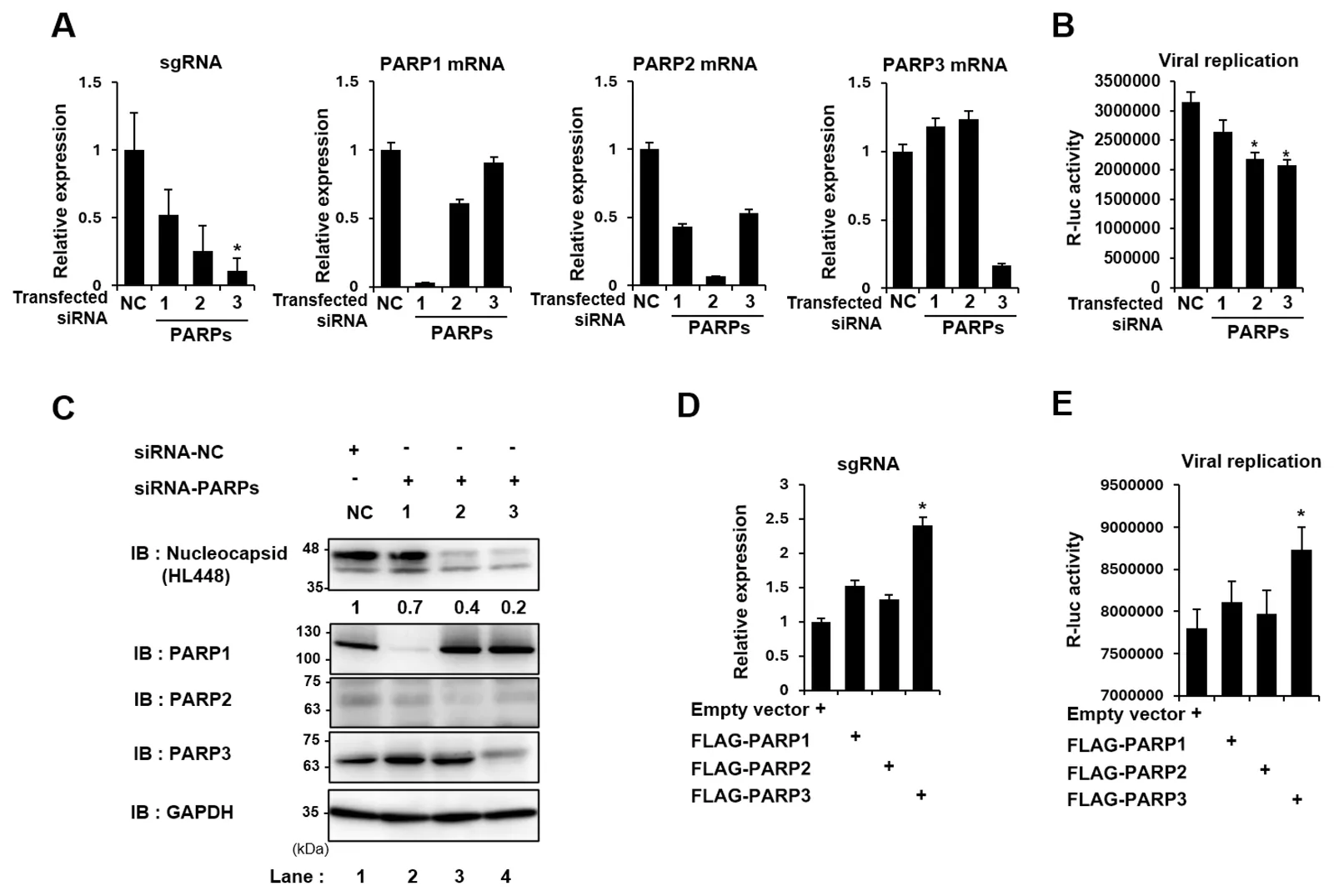
PARP3 promotes SARS-CoV-2 replicon replication in VeroE6/Rep3 cells **(A–C)** VeroE6/Rep3 reporter replicon cells were transfected with a non-targeting control siRNA (siRNA NC) or specific siRNAs targeting human PARP1, PARP2, or PARP3. **(A)** At 72 h after transfection, viral subgenomic RNA (sgRNA) levels were quantified by RT-qPCR, and **(B)** viral replication was measured by *Renilla* luciferase (R-luc) bioluminescence. Cell lysates were subjected to immunoblotting using the indicated antibodies. GAPDH served as the loading control. **(C)** The relative levels of nucleocapsid were quantified by densitometry and are indicated below each lane. IB, immunoblotting. **(D, E)** VeroE6/Rep3 cells were transfected with the empty vector pCAG-FLAG or with pCAG-FLAG-PARP1, pCAG-FLAG-PARP2, or pCAG-FLAG-PARP3. **(D)** Viral sgRNA transcription and **(E)** viral replication, as determined by *Renilla* luciferase activity, were measured at 72 h after transfection. Data represent the mean ± SEM from three independent experiments. *P < 0.05, compared with the controls. (unpaired two-tailed Student’s *t*-test).

**Fig. 2:**
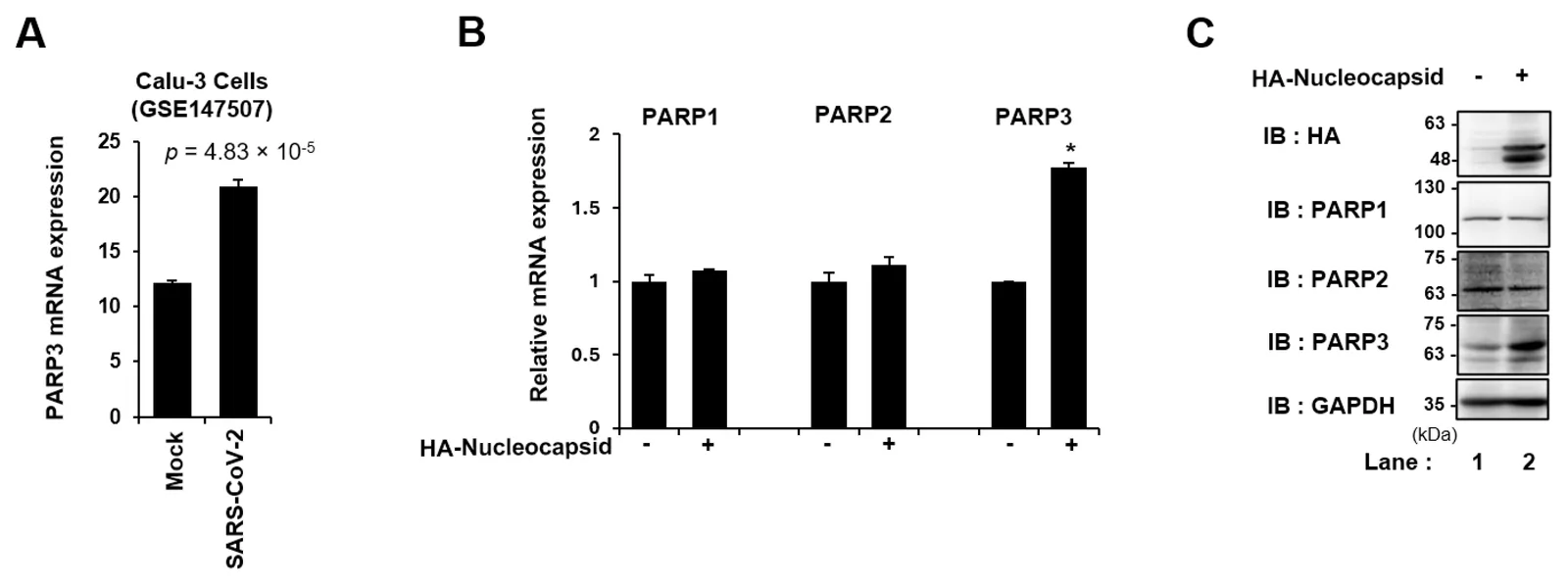
SARS-CoV-2 nucleocapsid enhances PARP3 expression **(A)** *In silico* transcriptomic analysis of RNA-sequencing data from Calu-3 cells using publicly available RNA-sequencing data (GSE147507), comparing mock and SARS-CoV-2-infected conditions. **(B, C)** HEK293T cells were transfected with pCAG-HA-nucleocapsid or an empty vector. At 48 h after transfection, the cells were harvested. **(B)** Relative mRNA expression levels of endogenous human PARP1, PARP2, and PARP3 were quantified by RT-qPCR. Data represent the means ± SEM from three biological replicates. Values for control cells were arbitrarily set to 1.0. *P < 0.05. (unpaired two-tailed Student’s *t*-test) **(C)** Immunoblot analysis of endogenous PARP1, PARP2, and PARP3 protein levels, HA-nucleocapsid expression, and GAPDH (loading control). IB, immunoblotting. Cell lysates were subjected to immunoblotting using the indicated antibodies. GAPDH served as the loading control.

**Fig. 3:**
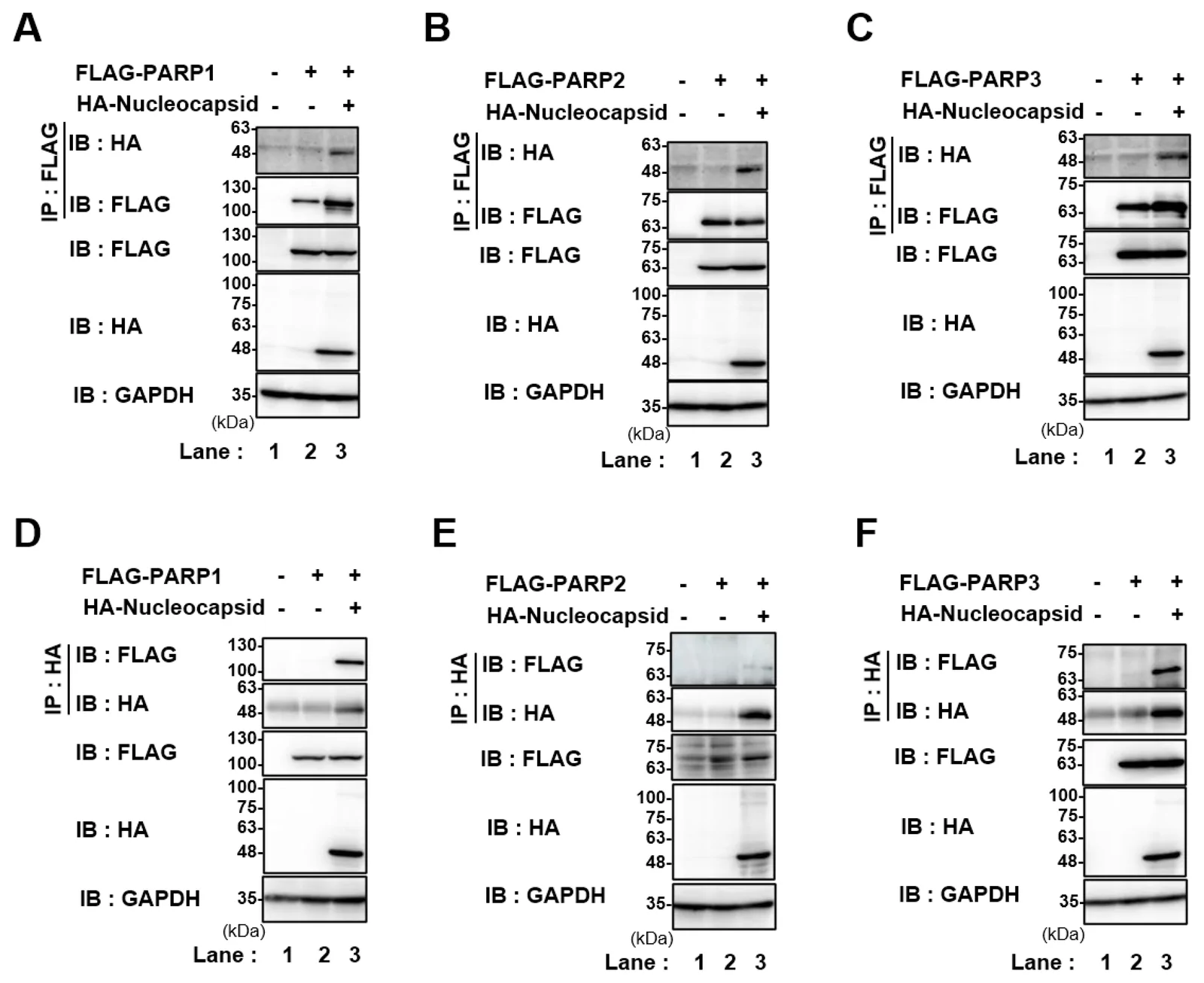
SARS-CoV-2 nucleocapsid associates with PARP1, PARP2, and PARP3 HEK293T cells were co-transfected with pCAG-HA-nucleocapsid and either pCAG-FLAG-PARP1, pCAG-FLAG-PARP2, or pCAG-FLAG-PARP3. At 48 h after transfection, cells were harvested. **(A–C)** Cell lysates were immunoprecipitated with anti-FLAG beads, followed by immunoblotting with anti-HA PAb (top panel) or anti-FLAG MAb (second panel). Input samples were immunoblotted with anti-FLAG MAb (third panel), anti-HA PAb (fourth panel), and anti-GAPDH MAb (fifth panel). **(D–F)** Cell lysates were immunoprecipitated with anti-HA PAb, followed by immunoblotting with anti-FLAG MAb (top panel) or anti-HA PAb (second panel). Input samples were immunoblotted with anti-FLAG MAb (third panel), anti-HA PAb (fourth panel), and anti-GAPDH MAb (fifth panel). IB, immunoblotting; IP, immunoprecipitation.

**Fig. 4:**
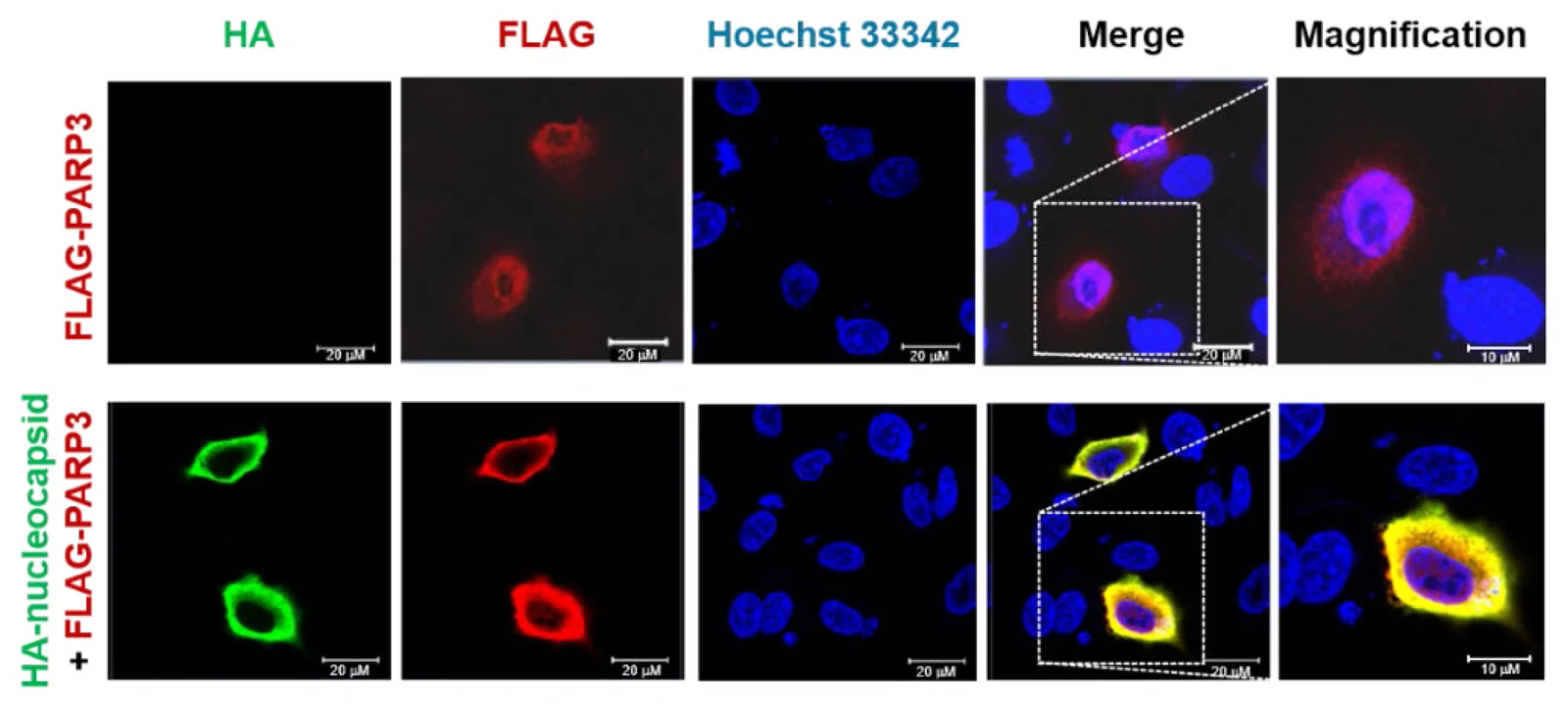
SARS-CoV-2 nucleocapsid colocalizes with PARP3 predominantly in the cytoplasm HEK293T cells were co-transfected with pCAG-FLAG-PARP3 and either pCAG-HA-nucleocapsid or an empty vector. At 48 h post-transfection, cells were fixed and subjected to indirect immunofluorescence staining using mouse anti-FLAG MAb and rabbit anti-HA PAb, followed by Alexa Fluor 594-conjugated goat anti-mouse IgG (red) and Alexa Fluor 488-conjugated goat anti-rabbit IgG (green), respectively. Nuclei were counterstained with Hoechst 33342 (blue). Scale bar, 20 μm. Merge, merged image of FLAG, HA, and Hoechst channels.

**Fig. 5:**
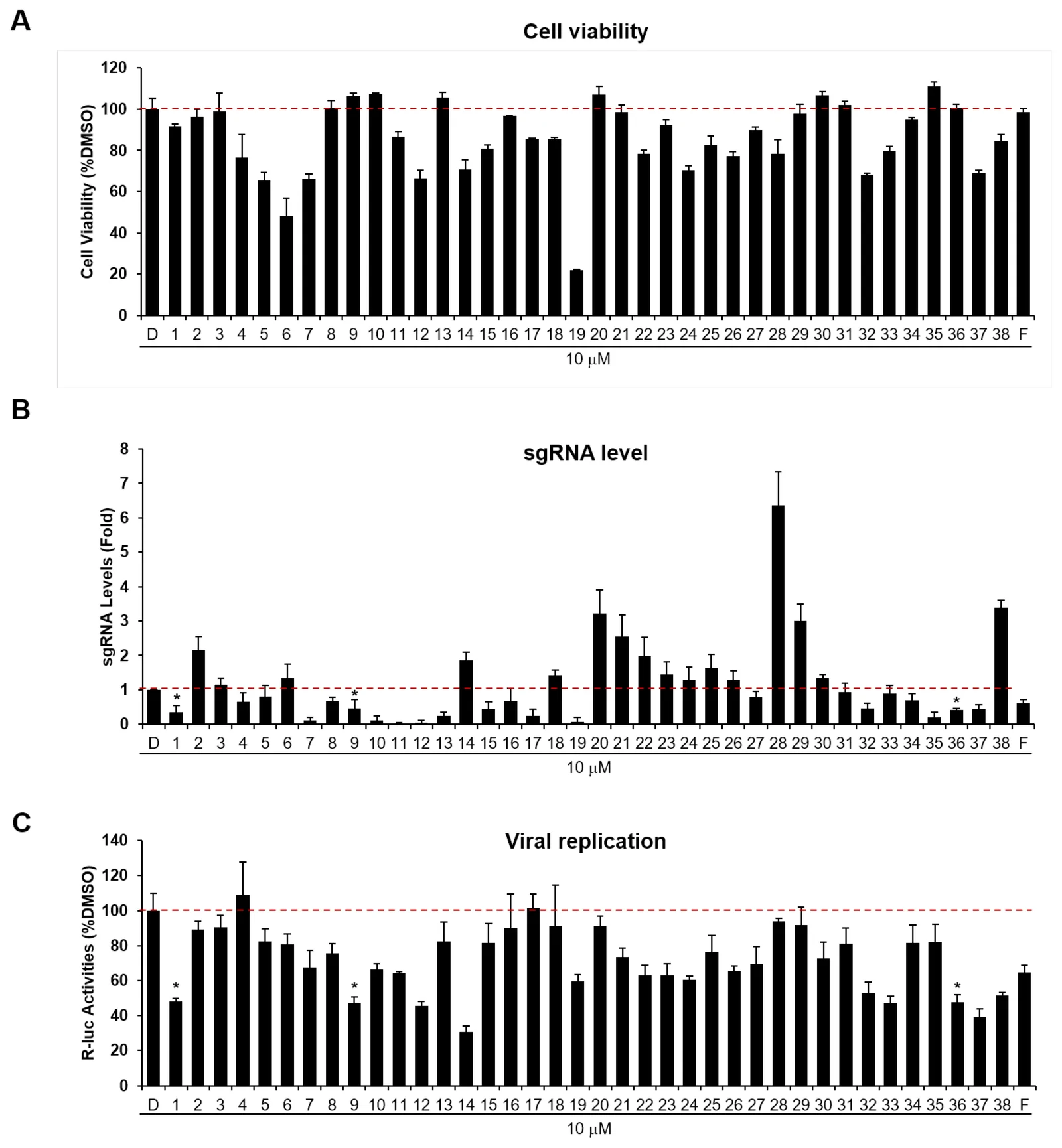
Screening of a PARP inhibitor library identifies compounds with anti-SARS-CoV-2 activity **(A)** VeroE6/Rep3 cells were treated with 38 PARP inhibitors at a concentration of 10 μM. At 72 h post-treatment, cell viability was assessed using CellTiter-Glo 2.0. D indicates the DMSO vehicle control, and F indicates the positive control favipiravir. **(B, C)** VeroE6/Rep3 reporter replicon cells were treated with 38 PARP inhibitors at a concentration of 10 μM. DMSO (D) and favipiravir (F) were included as vehicle and positive controls, respectively. **(B)** At 72 h after treatment, viral sgRNA levels were quantified by RT-qPCR, and **(C)** viral replication was assessed by *Renilla* luciferase (R-luc) bioluminescence. Values were normalized to the DMSO-treated control. Data represent the mean ± SEM from three biologically independent replicates. *P < 0.05 compared with the DMSO-treated control. Compound identities are listed in Table I. List of 38 poly(ADP-ribose) polymerase (PARP) inhibitors used in the primary screening assay.

**Fig. 6:**
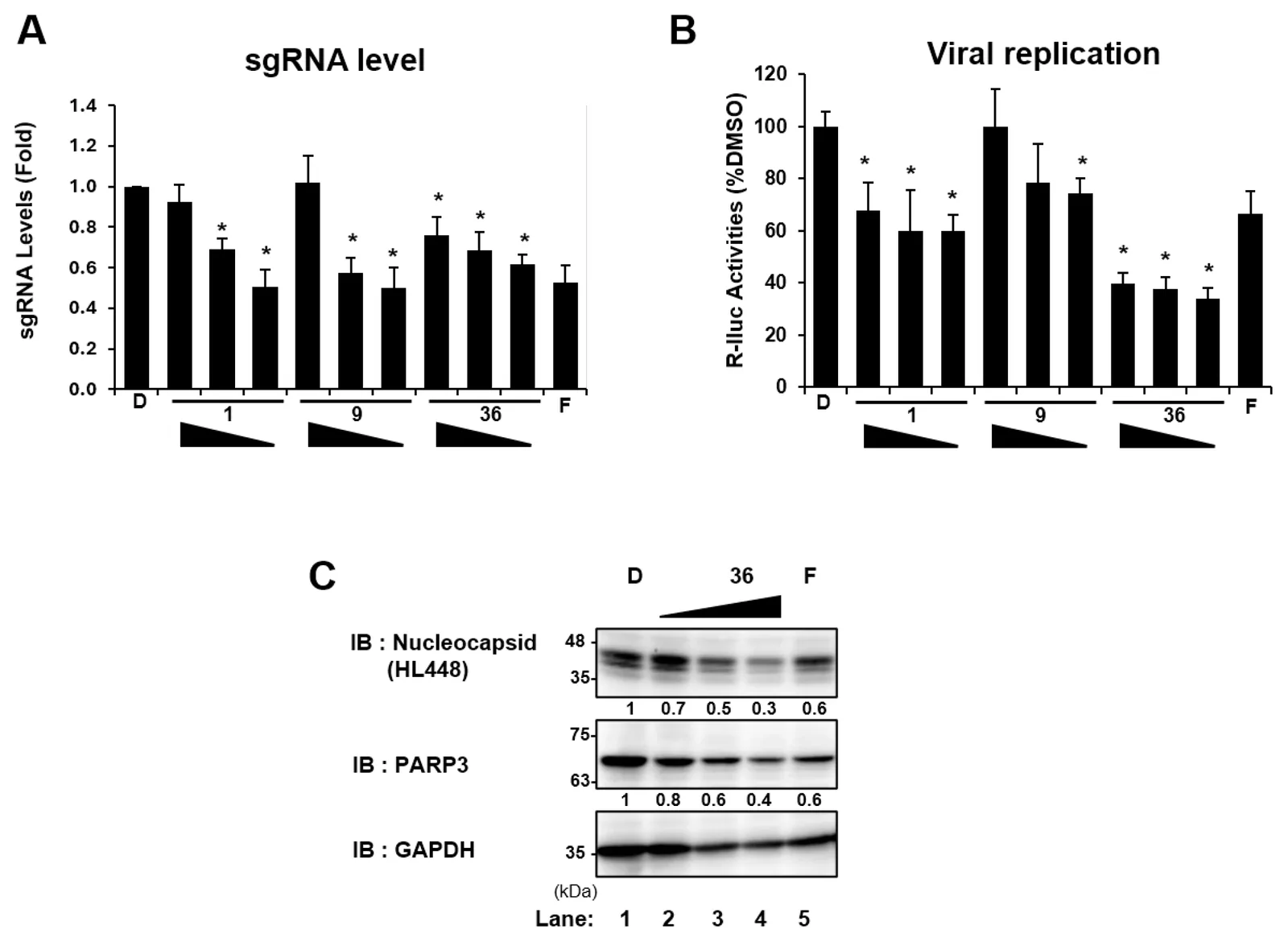
Venadaparib suppresses SARS-CoV-2 replication and reduces nucleocapsid expression **(A, B)** VeroE6/Rep3 reporter replicon cells were treated with three PARP inhibitors, niraparib (No. 1), veliparib (No. 9), or venadaparib (No. 36), at concentrations of 10, 1, 0.1 μM as indicated by black triangles. DMSO (D) and favipiravir (F) were used as vehicle and positive controls, respectively. **(A)** At 72 h after treatment, viral sgRNA levels were quantified by RT-qPCR, **(B)** viral replication was assessed by *Renilla* luciferase (R-luc) bioluminescence, and **(C)** VeroE6/Rep3 reporter replicon cells were treated with venadaparib (No. 36) at concentrations of 0.1, 1, or 10 μM as indicated by black triangle. Cell lysates were subjected to immunoblotting using the indicated antibodies. GAPDH served as the loading control. Data in (A) and (B) represent the mean ± SEM from three biologically independent replicates. *P < 0.05 compared with the DMSO-treated control.

**Fig. 7:**
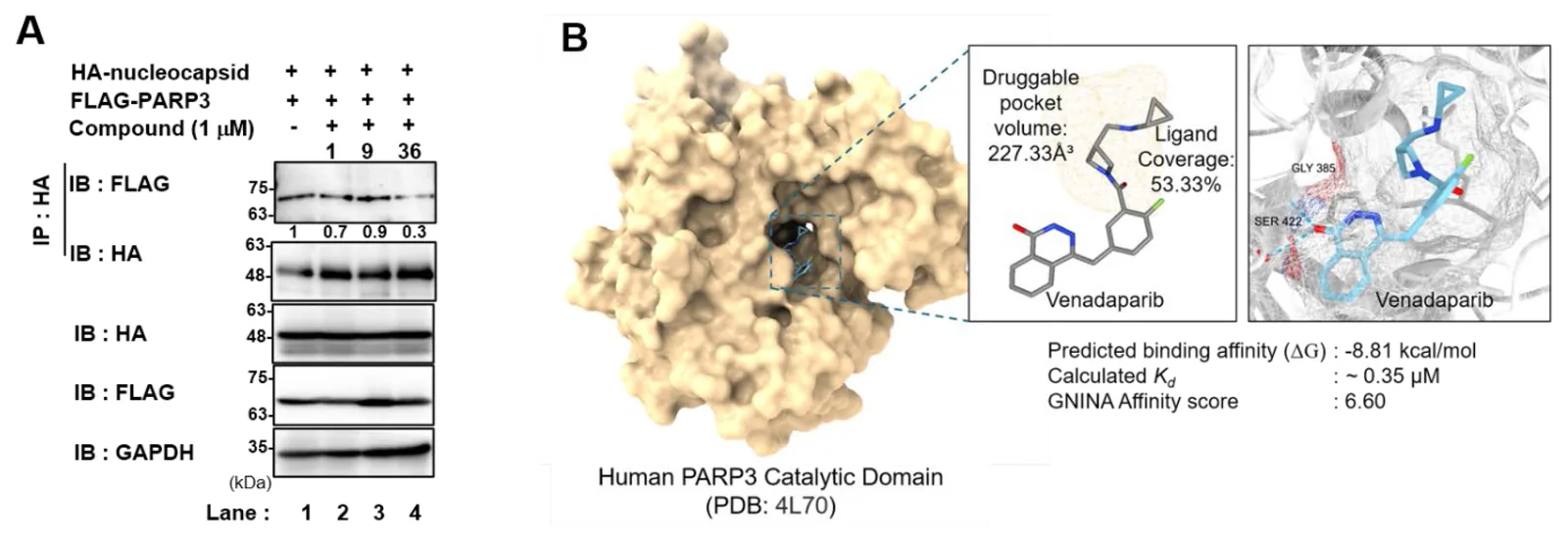
Venadaparib reduces the association between PARP3 nucleocapsid and is predicted to bind to the PARP3 catalytic domain **(A)** HEK293T cells were co-transfected with pCAG-HA-nucleocapsid and pCAG-FLAG-PARP3. At 24 h after transfection, cells were treated with 1 μM niraparib (No. 1), veliparib (No. 9), or venadaparib (No. 36). At 72 h after treatment, cell lysates were immunoprecipitated with anti-HA PAb and subjected to immunoblotting using the indicated antibodies. The relative levels of immunoprecipitated FLAG-PARP3 were quantified by densitometry and are indicated below each lane. **(B)** Molecular docking analysis of venadaparib with the human PARP3 catalytic domain (PDB ID: 4L70) using DiffDock. The predicted binding conformation of venadaparib within the PARP3 catalytic pocket is shown. Druggable pocket volume and ligand coverage were calculated using DoGSite3. Hydrogen bonds with Gly385 and Ser422 are indicated. Binding affinity was evaluated using the GNINA scoring algorithm (predicted binding affinity (Δ*G*) = -8.81 kcal/mol). The compound occupied 53.33% of the druggable subpocket volume as defined by DoGSite3.

**Fig. 8:**
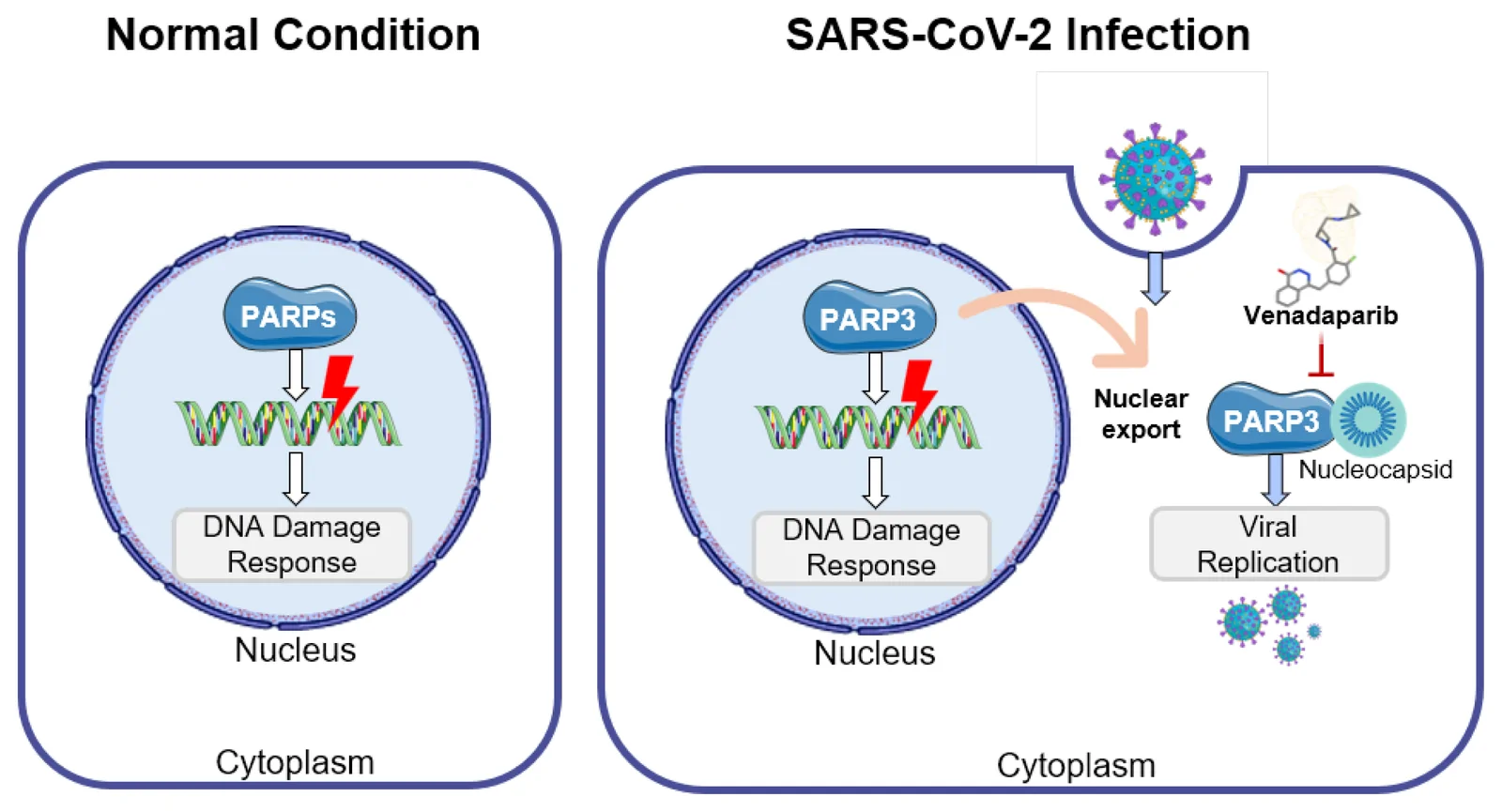
A proposed working model for the role of PARP3 in SARS-CoV-2 replication via interaction with the viral nucleocapsid protein SARS-CoV-2 nucleocapsid promotes the cytoplasmic redistribution of PARP3. Cytoplasmic PARP3 interacts with the viral nucleocapsid (N) protein, potentially facilitating viral replication in the cytoplasm. The PARP inhibitor venadaparib reduces the PARP3–nucleocapsid association, which may contribute to the suppression of SARS-CoV-2 replication.

**Table I:** Compound library

No.	Compound Name	No.	Compound Name
1.	Niraparib (MK-4827)	20.	Berberine Chloride
2.	UPF 1069	21.	Picolinamide
3.	BYK204165	22.	JW55
4.	AZD5305	23.	Benzamide
5.	Rucaparib Camsylate	24.	RK-287107
6.	Rucaparib	25.	RBN012759
7.	Talazoparib (BMN673)	26.	WIKI4
8.	ME0328	27.	NVP-TNKS656
9.	Veliparib	28.	G007-LK
10.	Olaparib (AZD2281)	29.	NU1025
11.	Niraparib (MK-4827) tosylate	30.	BGP-15 2HCl
12.	Stenoparib (E7449)	31.	GeA-69
13.	A-966492	32.	MN64
14.	Pamiparib (BGB-290)	33.	AZD-2461
15.	Iniparib (BSI-201)	34.	PJ34 HCl
16.	Hydroxyquinazoline	35.	3-Aminobenzamide
17.	NMS-P118	36.	Venadaparib (IDX-1197)
18.	AG-14361	37.	XAV-939
19.	HI-TOPK-032	38.	Fluzoparib

List of 38 poly(ADP-ribose) polymerase (PARP) inhibitors used in the primary screening assay.
